# Revealing potential Rab proteins participate in regulation of secretory autophagy machinery

**DOI:** 10.1002/kjm2.12848

**Published:** 2024-05-28

**Authors:** Pei‐Wen Lin, Man‐Ling Chu, Yu‐Wen Liu, Yu‐Cing Chen, Yao‐Hsiang Shih, Sheng‐Hui Lan, Shang‐Ying Wu, I‐Ying Kuo, Hong‐Yi Chang, Hsiao‐Sheng Liu, Ying‐Ray Lee

**Affiliations:** ^1^ Master of Science Program in Tropical Medicine, College of Medicine Kaohsiung Medical University Kaohsiung Taiwan; ^2^ Department of Microbiology and Immunology College of Medicine, National Cheng Kung University Tainan Taiwan; ^3^ Department of Anatomy School of Medicine, College of Medicine, Kaohsiung Medical University Kaohsiung Taiwan; ^4^ Department of Life Sciences and Institute of Genome Sciences National Yang Ming Chiao Tung University Taipei Taiwan; ^5^ Department of Microbiology and Immunology School of Medicine, College of Medicine, Taipei Medical University Taipei Taiwan; ^6^ Department of Biotechnology College of Life Science, Kaohsiung Medical University Kaohsiung Taiwan; ^7^ Center for Cancer Research Kaohsiung Medical University Kaohsiung Taiwan; ^8^ Teaching and Research Center, Kaohsiung Municipal Hsiao‐Kang Hospital Kaohsiung Taiwan; ^9^ Department of Microbiology and Immunology College of Medicine, Kaohsiung Medical University Kaohsiung Taiwan; ^10^ Faculty of Post‐Baccalaureate Medicine, College of Medicine, Kaohsiung Medical University Kaohsiung Taiwan; ^11^ Center for Tropical Medicine and Infectious Disease, Kaohsiung Medical University Kaohsiung Taiwan

**Keywords:** Rab protein, secretory autophagy, unconventional protein secretion

## Abstract

Autophagy can be classified as degradative and secretory based on distinct functions. The small GTPase proteins Rab8a and Rab37 are responsible for secretory autophagy‐mediated exocytosis of IL‐1β, insulin, and TIMP1 (tissue inhibitor of 54 metalloproteinase 1). Other Rab family members participating in secretory autophagy are poorly understood. Herein, we identified 26 overlapped Rab proteins in purified autophagosomes of mouse pancreatic β‐cell “Min‐6” and human lung cancer cell “CL1‐5‐Q89L” with high secretory autophagy tendency by LC–MS/MS proteomics analysis. Six Rab proteins (Rab8a, Rab11b, Rab27a, Rab35, Rab37, and Rab7a) were detected in autophagosomes of four cell lines, associating them with autophagy‐related vesicle trafficking. We used CL1‐5‐Q89L cell line model to evaluate the levels of Rab proteins colocalization with autophagy LC3 proteins and presence in purified autophagosomes. We found five Rab proteins (Rab8a, Rab11b, Rab27a, Rab35, and Rab37) are highly expressed in the autophagosome compared to the normal control by immunoblotting under active secretion conditions. However, only Rab8a, Rab35, and Rab37 showing high colocalization with LC3 protein by cofocal microscopy. Despite the discrepancy between the image and immunoblotting analysis, our data sustains the speculation that Rab8a, Rab11b, Rab27a, Rab35, and Rab37 are possibly associated with the secretory autophagy machinery. In contrast, Rab7a shows low colocalization with LC3 puncta and low level in the autophagosome, suggesting it regulates different vesicle trafficking machineries. Our findings open a new direction toward exploring the role of Rab proteins in secretory autophagy‐related cargo exocytosis and identifying the cargoes and effectors regulated by specific Rab proteins.

## 1 INTRODUCTION

Molecular secretion is important for interaction with mammalian cells in the tissue microenvironment. A conventional endoplasmic reticulum (ER)‐to‐Golgi membrane pathway comprises multistep steps, including vesicle synthesis, cargo loading, concentration, processing, vesicle transport, targeting, docking, and plasma membrane fusing.^1^ The proteins endowed with N‐terminal signal peptides are secreted from the cells through the conventional secretion pathway. In contrast, proteins without N‐terminal signal peptides are delivered out of cells through the unconventional protein secretion (UPS) pathway.^2^, ^3^ Four nonvesicular and vesicular UPS pathways have been reported. The former contains type I: protein direct translocation across the cellular membrane, and type II: ABC‐transporter‐based secretion. The latter consists of type III, the autophagy‐based secretion; type IV, proteins bypass the Golgi complex for trafficking to the cellular membrane.^2^, ^3^

Autophagy is a process that maintains cellular homeostasis by controlling intracellular biomass through the traditional degradative autophagy pathway to protect cells from harmful stress. In the conventional autophagy pathway, the serine/threonine kinase ULK1 and Beclin‐1 combined with autophagy‐related protein 14 (Atg14) and type III phosphatidylinositol 3‐kinase (PI3K) Vps34 together with other Atg proteins result in the formation of double‐membrane autophagosome (AP), which recruits cargos including aggregated proteins, damaged organelles, pathogens, and microRNAs in the cytoplasm in cooperation with cargo receptors such as p62/SQSTM1 followed by fusion with the lysosome for degradation.^4^ In contrast to the degradative autophagy pathway, cells may undergo the secretion of cytoplasmic constituents instead of their degradation, named secretory autophagy (or unconventional secretory autophagy pathway) through a shared but partially divergent pathway.^5^ The recruited cargos of the secretory autophagy are transported to autophagic‐like vesicles and exported to the extracellular environment.^5^, ^6^ IL‐1β, IL‐8, and HMGB1 do not contain a secretion signal peptide, and their exocytosis is triggered by inflammasome activation followed by secretory autophagy.^5^, ^7^ In addition, secretory autophagy is not only restricted to inflammasome substrate release but also exerts the release of leaderless cytosolic proteins, including galectin‐3, annexin‐1, and tubulin with autophagic‐dependent secretome.^5^, ^8^ Moreover, it also contributes to microbial release and transmission from cells.^9^, ^10^ In summary, secretory autophagy plays important roles in unconventional protein exocytosis, cell‐to‐cell communication, and microbial transmission. However, the factors regulating autophagic vesicle biogenesis, trafficking, and cargo release remain poorly understood.

Rab family small GTPases are key regulators of intracellular membrane trafficking in eukaryotes.^11^ Multiple Rabs, including Rab1, Rab5, Rab7, Rab8b, Rab9, Rab24, and Rab33, are involved in the canonical degradative autophagy machinery.^12^ Rab8a regulates polarized sorting to the cellular membrane and is associated with the flow of synthesized proteins to the basolateral surface of cells.^13^ It has also been demonstrated to participate in the autophagic secretion of IL‐1β.^7^ However, Rab8b, an isoform of Rab8a, exerts its function on the degradative autophagy machinery.^14^ Rab7, Rab32, Rab33b, and Rab34 are known to be involved in AP maturation.^14^, ^15^, ^16^ Rab11 is the first Rab GTPase discovered to regulate exosome secretion, and it regulates the fusion of APs with multivesicular bodies (MVBs).^17^, ^18^ In the MVB‐mediated pathway, Rab11 recruits MVB and fuses with APs to form amphisomes. Rab8a and Rab27a are involved in the transport of amphisome to the plasma membrane.^19^ Rab24 functions in the clearance of autolysosomes under basal conditions.^20^ Rab37 regulates cargo exocytosis by cycling between an inactive GDP‐bound form and an active GTP‐bound form, and plays a regulatory role in secretory autophagy for insulin and TIMP1 secretion.^6^, ^21^, ^22^ In summary, Rab family proteins seem to play a determinant role in vesicle trafficking toward either the degradative or the secretory autophagy machinery. Rab proteins are key regulators responsible for intracellular trafficking of vesicles, including exosomes, MVBs (multivesicular bodies), amphisomes and lysosomes and summarized as the Table 1. Nevertheless, the understanding of the roles of Rab proteins in secretory autophagy and related cargo exocytosis remains in its infant stage. Therefore, this study focuses on revealing the potential Rab proteins involved in secretory autophagy.

**TABLE 1:** Rab proteins cooperate to regulate secretory autophagy.

Rab protein	Function‐related to secretory autophagy
Rab8a	Rab8a is required for autophagy‐mediated IL‐1β secretion pathway under starvation and inflammasome activation.^7^
Rab11	Rab11 mediates MVBs fusing with autophagosomes to form amphisome followed by exocytosis.^19^
Rab27a	Rab27a together with Rab8a are responsible for transporting the amphisome to the plasma membrane. Cargoes including Wnt5A, MMP2, IL‐6, IL‐8, annexin A2, galectin‐1, HMGB1, type I collagen and fibronectin, are delivered through the MVB‐dependent secretory autophagy.^19^
Rab37	RAB37 dependent secretory autophagy participates in TIMP1 secretion in lung cancer both *in vitro* and *in vivo*.^22^ RAB37 dependent secretory autophagy promotes insulin secretion to maintain the homeostasis of insulin and glucose both *in vitro* and *in vivo*.^6^

Abbreviations: HMGB1, high mobility group box 1; MMP2, matrix metalloproteinase 2; MVE, multivesicular endosome; TIMP1, tissue inhibitor of 54 metalloproteinase 1.

## 2 MATERIALS AND METHODS

### 2.1 Cell lines and cell culture

CL1‐5 cells, a human lung adenocarcinoma cell line, transfected with blank vector and active‐form Rab37 (Q89L) construction to generate CL1‐5 vector cell line and CL1‐5‐Q89L cell line, and were obtained from Dr. Yi‐Ching Wang's lab (National Cheng Kung University, Tainan, Taiwan). The human hepatoma cell line (Hep3B) and the colon cancer cell line (SW480) were purchased from Bioresource Collection and Research Center (Hsinchu, Taiwan). In addition, Min‐6, an insulinoma cell line, was a kind gift from Dr. Jun‐ichi Miyazaki (Osaka University, Osaka, Japan). These cells were maintained in Dulbecco's modified Eagle's medium (DMEM, Gibco, Grand Island, NY, USA) with 10% fetal bovine serum (Gibco) and penicillin/streptomycin (Gibco) at 37°C in a 5% CO_2_ incubator.

### 2.2 AP induction and accumulation

Starvation was used to induce cellular autophagy for 24 h, and the cells were treated with 25 μM chloroquine (CQ; Sigma, St. Louis, MO, USA) to block autophagic flux (degradative autophagy) and causing AP accumulation.

### 2.3 AP purification

The cells were suspended in the hypotonic buffer and homogenized by passing through a 32G needle 30 times. The homogenate was diluted with homogenization buffer (HB) with 0.5 mM glycyl‐l‐phenylalanine 2‐naphthylamide (GPN) and 1% DMSO and incubated for 7 min at 37°C. The homogenate was centrifuged at 4000 rpm for 5 min to separate the post‐nuclear supernatant (PNS) and the nucleus. The PNS was centrifuged at 28000 rpm with different concentrations of Nycodenz (Axis‐Shield, Dundee DD2 iXA, Scotland, UK) gradient overnight, and the gradients were divided into three fractions. The fraction containing AP and endoplasmic reticulum was isolated and separated for next centrifugation at 20000 rpm overnight with Percoll (GE Healthcare, Chalfont St Giles, Buckinghamshire, UK) and Nycodenz gradient. The AP fraction was isolated and separated for further centrifugation with Optiprep (STEMCELL, Vancouver, BC, Canada) gradient at 20000 rpm for 1 h. The AP band was collected for final centrifugation with HB to isolate the AP pellet. The extraction was performed as previously reported.^23^

### 2.4 In solution digestion and LC–MS/MS analyses

The protein was extracted from the APs of various cell lines. Protein extracts were denatured and then alkylated in dithiothreitol (7 mM) and iodoacetamine (21 mM), respectively, at 37°C for 30 min. Proteins were digested by trypsin (Sigma, T4049) at 37°C for 16 h. The peptide mixtures were separated on a 3 × 150 mm C18 column (Gemini, Phenomenex, 00F‐4435‐E0) coupled to a high‐performance liquid chromatography system (Beckman Coulter, CA, USA) using an acetonitrile gradient in 0.1% ammonium hydroxide solution, with approximately 30 fractions. The peptide fractions were analyzed on a nanoLC‐Q ExactiveTM HF mass spectrometer (Thermo Fisher, San Jose, USA) equipped with an HPLC system (M Class, Waters, MA, USA). MS raw files were uploaded into Proteome Discoverer (version 2.1, Thermo Fisher, MA, USA) with the default setting to generate peak lists for protein identification using the MASCOT search engine (version 2.5, Matrix Science, MA, USA) against the Swiss‐Prot Mus musculus protein database (released in Jan. 2016). The peptides sharing an identical sequence among multiple proteins were assigned to the one with the highest protein score. Identifying peptides and proteins with a false discovery rate of less than 1% was considered acceptable.

### 2.5 Immunofluorescence assay

The cells were fixed with 3.7% formaldehyde (Sigma) for 30 min and incubated in 0.1% Triton X‐100 (Sigma) at RT for 30 min after treatment. After washing with PBS, cells were incubated with specific primary antibodies (LC3, MBL, Minato‐ku, Tokyo, Japan; Rab7a, GeneTex, Hsinchu City, Taiwan; Rab8a, Proteintech, Rosemont, IL, USA; Rab11b, GeneTex; Rab27a, Proteintech; Rab35, GeneTex; Rab37, Proteintech) at 4°C overnight. Cells were incubated with anti‐rabbit Alexa Fluor® 594‐conjugated secondary antibody (Invitrogen, Waltham, MA, USA) and anti‐mouse Alexa Fluor® 488‐conjugated secondary antibody (Invitrogen) at room temperature (RT) for 1 h. Fluoroshield™ with DAPI (Sigma) was used for nucleus staining and mounting. The fluorescent signal was detected using a confocal laser scanning microscope (LSM 700, Zeiss, Jena, Germany). To prevent analysis bias, we used the semiautomatic analysis. The methods are provided by the co‐author Dr. Yao‐Hsiang Shih, KMU. The processing code was scripted by the Fiji integrated development environment in ImageJ macro language. Briefly, we gathered the cell area and location through the threshold function first, then used the same function to calibrate and extract the autophagy puncta from the original image (min gray value set to the modal gray value plus two‐fold of histogram standard deviation; max gray value reduces the histogram standard deviation as new max gray value.). After that, we used Mander's colocalization to determine the Rab protein‐positive AP colocalization ratio.

### 2.6 Immunoblotting analysis

Cells were lysed with lysis buffer, and the protein concentration was quantified by Bradford Protein Assay Kit (Thermo Scientific, Waltham, MA, USA). The cell lysate was separated by 12% SDS‐polyacrylamide gel followed by transfer to a PVDF membrane (Millipore, Billerica, MA, USA) in transfer buffer at 100 V for 1.5 h using an electroblotter (Amersham Pharmacia Biosciences Corp., NJ, USA). The transferred membrane was blocked with 5% milk in PBST buffer for 1 h at RT. The membrane was incubated with the primary antibody (Rab7a, GeneTex; Rab8a, Proteintech; Rab11b, GeneTex; Rab27a, GeneTex; Rab35, GeneTex; Rab37 from Dr. Yi‐Ching Wang's lab; LC3, MLB) at 4°C overnight. The membrane was incubated with an HRP‐conjugated secondary antibody (Invitrogen) at RT for 1 h, and the enhanced chemiluminescence reagent (Millipore) was used to evaluate the protein bands. Three independent assays were performed, and one of the results was shown. Densitometry analysis was performed with ImageJ v1.53t.

### 2.7 Statistical analysis

Data are presented as the mean ± SD of separate experiments. Differences between the test and control groups were analyzed using one‐way ANOVA and Fisher's least significant difference test. A *p*‐value of <0.05 was considered statistically significant in all tests.

## 3 RESULTS

### 3.1 Identification of Rab proteins associated with secretory autophagy in the APs of two cell lines under secretory autophagy conditions

**FIGURE 1:**
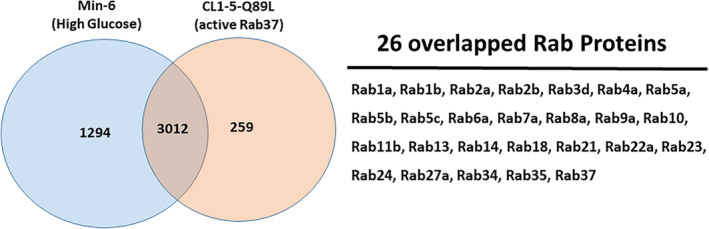
Identification of 26 Rab proteins in the purified autophagosomes (APs) of mouse Min‐6 and human lung cancer CL1‐5‐Q89L under stimulation of secretory autophagy. The proteins in the purified APs of Min‐6 and CL1‐5‐Q89L cells were analyzed by LC–MS/MS proteomics analysis. We identified 26 Rab family proteins in 3012 overlapped proteins between Min‐6 and CL1‐5‐Q89L cells.

Rab family proteins participating in the vesicle trafficking to transport proteins within the cell have been reported. Various Rab proteins are known to regulate the autophagy machinery, including the degradative and secretory pathways. However, the Rab proteins modulating the secretory autophagy machinery are poorly studied. Rab8a is the first Rab protein reported to participate in secretory autophagy.^7^ We reveal that activated Rab37 promotes the secretion of insulin in mouse β cells (Min‐6) and TIMP1 in human lung cancer cells (CL1‐5‐Q89L, harboring active Rab37), respectively.^6^, ^22^ Herein, we further purified the APs from mouse Min‐6 and human lung cancer CL1‐5‐Q89L cells under active secretory conditions and analyzed the proteins within by LC–MS/MS proteomics analysis. Twenty six Rab family proteins were identified in 3012 overlapped proteins between these two cell lines (Figure 1). Among these 26 Rab proteins, six showed autophagy‐related functions and were detected in the purified APs of four cell lines (mouse Min‐6, human Hep3B, SW480, and CL1‐5‐Q89L cells) (Table 2). To clarify whether these Rab proteins participate in the secretory autophagy machinery, we utilized the lung cancer cell line CL1‐5‐Q89L, which overexpresses active Rab under starvation conditions, as a model to induce the secretory autophagy.

**TABLE 2:** Six autophagy‐related Rab proteins identified in the purified autophagosomes of four cell lines.

Mouse	Human			Function‐related to autophagy
Min‐6	Hep3B	SW480	CL1‐5‐Q89L	
Rab7a	Rab7a	Rab7a	Rab7a	Autophagosome maturation^24^
Rab8a	Rab8a	Rab8a	Rab8a	Autophagy‐based secretion^7^, ^25^
Rab11b	Rab11b	Rab11b	Rab11b	A critical regulator of PAR1 trafficking^26^
Rab27a	Rab27a	Rab27a	Rab27a	MVE docking at the plasma membrane and size of MVEs^27^
Rab37	Rab37	Rab37	Rab37	Promote autophagosome formation^28^
Rab35	Rab35	Rab35	Rab35	Apical endocytic recycling^29^

Abbreviations: MVE, multivesicular endosome; PAR1, protease‐activated receptor‐1.

### 3.2 Colocalization of six autophagy‐related Rab proteins with autophagy LC3 protein in CL1‐5‐Q89L and CL1‐5 vector cells under starvation conditions

**FIGURE 2:**
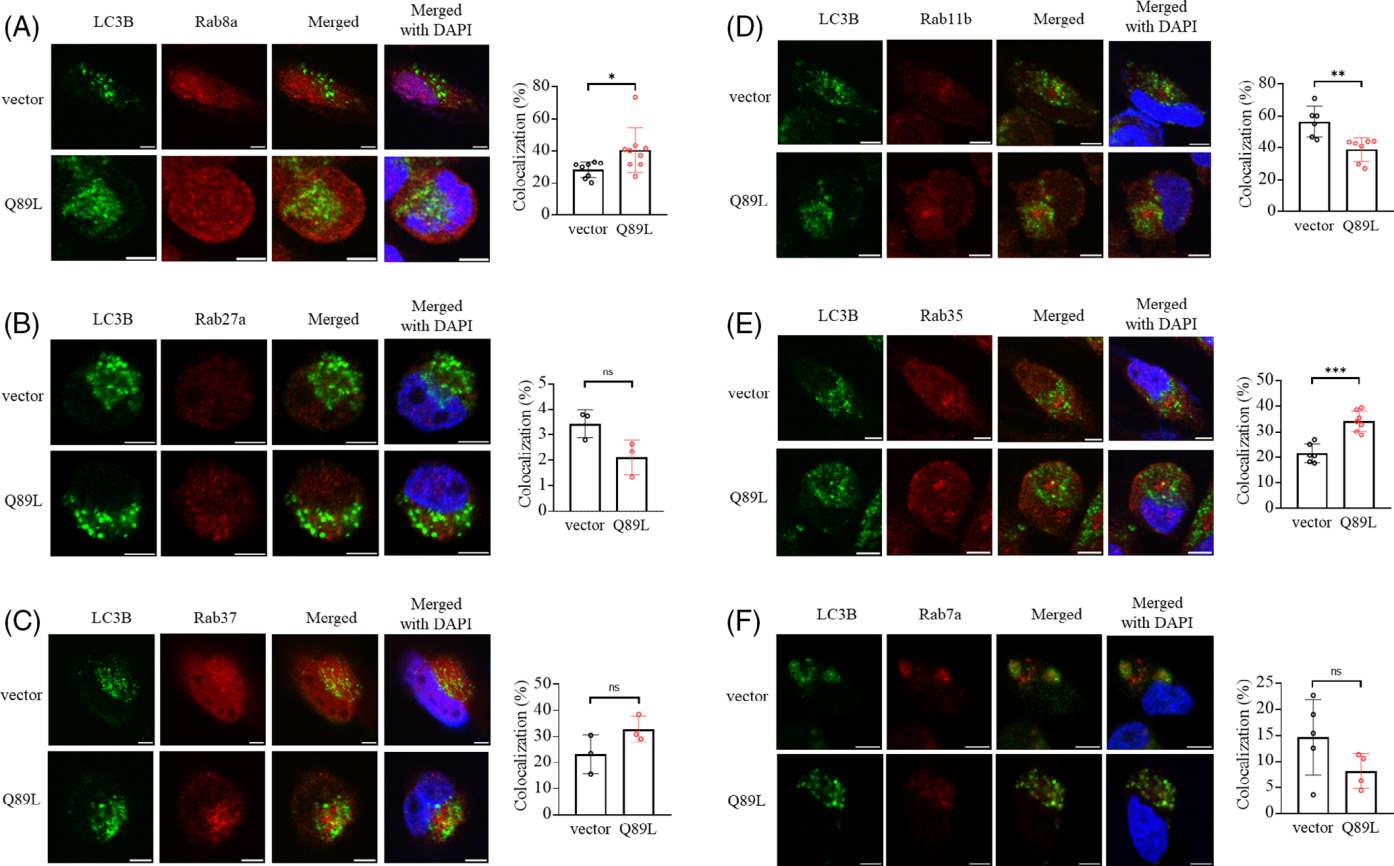
Colocalization of six autophagy‐related Rab and LC3 proteins in lung cancer CL1‐5 and CL1‐5‐Q89L cells under active secretory conditions. The cells were immunostaining with FITC‐conjugated anti‐LC3 antibody (green) or rhodamine‐conjugated anti‐Rab antibodies (red): (A) Rab8a, (B) Rab27a, (C) Rab37, (D) Rab11b, (E) Rab35, and (F) Rab7a at 24 h after starvation conditions. Scale bar = 5 μm. Green LC3 puncta represent autophagosomes, and red puncta represent Rab‐harbored vesicles. The colocalization of LC3 and Rab proteins was shown as a yellow color. For quantification, yellow immunofluorescence intensity was divided by red intensity for groups A, D, and E and green intensity for groups B, C, and F to calculate the percentage of LC3‐Rab colocalization. DAPI staining represents nuclei. ns: no significance, *: *p* < 0.05, **: *p* < 0.01, ***: *p* < 0.001.

We already confirmed that either high glucose or serum starvation could activate Rab37 and induce secretory autophagy to promote exocytosis of insulin from β‐cell and TIMP1 from lung cancer cells.^6^, ^21^, ^22^ We hypothesize that under serum starvation conditions together with overexpression of active‐form Rab37 (Q89L), secretory Rab‐anchored vesicles will fuse with autophagic vesicles in CL1‐5‐Q89L cells (Q89L) compared to CL1‐5 control cells (vector). We investigated the association of Rab and LC3 proteins (representing AP) by measuring the colocalization (yellow punctum) of six Rab and LC3 proteins using FITC‐conjugated anti‐LC3 antibody (green) and rhodamine‐conjugated anti‐Rab antibodies (red) in CL1‐5 (vector) and CL1‐5‐Q89L (Q89L) cells. Notably, we observed that only Rab8a, Rab35, and Rab37 showed higher colocalized Rab protein and LC3 puncta in Q89L cells compared to vector control cells (Figure 2A,C,E). In contrast, Rab27a, Rab11b, and Rab7a showed lower colocalization with LC3 puncta in the Q89L cells than in vector control cells (Figure 2B,D,F).

### 3.3 The expression levels of six autophagy‐related Rab proteins in the purified APs of CL1‐5‐Q89L and CL1‐5 cells under starvation conditions

**FIGURE 3:**
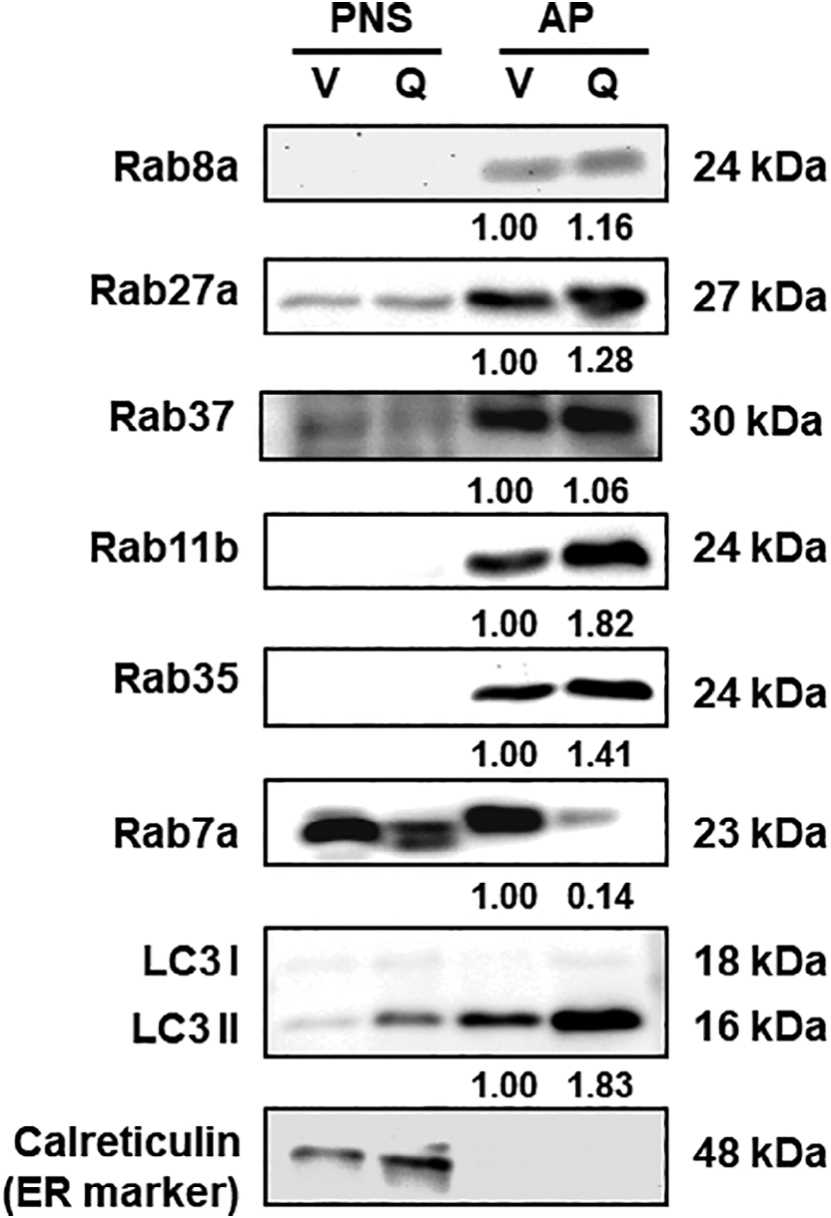
The levels of six autophagy‐related Rab proteins in the purified autophagosomes (APs) of lung cancer CL1‐5 (V) and CL1‐5‐Q89L (Q) cells under active secretory conditions. We compared six autophagy‐related Rab proteins in the post‐nucleus supernatant (PNS) and AP fractions of CL1‐5 (V) and CL1‐5‐Q89L (Q) cells under serum starvation conditions by immunoblotting using specific antibodies. LC3II is the marker of the AP, and calreticulin is the marker of ER. The numbers under the protein bands represent fold changes in protein levels compared to the control (AP‐V). An equal amount of protein (30 μg/well) from each fraction was loaded into the gel. V: CL1‐5 vector control: harboring the vector plasmid; Q: CL1‐5‐Q89L: harboring active‐form Rab37 plasmid.

To further confirm our notion, the APs of CL1‐5‐Q89L and CL1‐5 vector control cells under active secretory conditions (starvation) were purified following our previous protocol.^30^ High levels of calreticulin (representing ER) in the post‐nucleus supernatant (PNS) fraction, together with a high level of LC3II (representing AP vesicles) in the AP fraction, demonstrated the purity of the extracted APs (Figure 3). Under such conditions, the levels of the above six Rab proteins in the AP of CL1‐5‐Q89L (high secretory autophagy conditions) and CL1‐5 (low secretory autophagy conditions) cells were compared by immunoblotting analysis. We expect that the levels of secretion‐related Rab proteins will be higher in the APs (AP‐Q; active‐form Rab37) compared to vector control (AP‐V; vector only) as well as the post‐nucleus supernatant (PNS‐V and PNS‐Q). Our data showed: (1) the levels of Rab8a, Rab27a, Rab37, Rab11b, and Rab 35 proteins are higher in the AP fractions compared to the PNS fractions (Figure 3; AP vs. PNS); (2) in the AP fraction, these five Rab protein levels are higher in the cells harboring active form Rab37 (AP‐Q) compared to vector control (AP‐V) (Figure 3; AP‐V vs. AP‐Q). Among these five Rab proteins (Rab8a, Rab27a, Rab37, Rab11b, and Rab 35), Rab8a, Rab27a, and Rab37 participate in secretory autophagy have been reported,^7^, ^19^ which is consistent with our speculation that higher Rab protein levels detected in the APs may participate in the secretory autophagy under active secretory conditions. Therefore, we predicate that Rab11b and Rab35 are also potential secretory autophagy‐related proteins. In contrast, Rab7a level was very low in the secretory‐orientated APs (Figure 3, row 6) and low colocalization with LC3 in Figure 2F, indicating that it is not involved in the secretory autophagy progression.

Taken together, the data of confocal image analysis in Figure 2 and the immunoblotting in Figure 3 are partially consistent. The possible reason of Rab11b and Rab27a‐anchored vesicles showing low colocalization with APs is that they may be located very close to each other or form complexes with other proteins in the cells to execute their secretory functions. Further exploration is required to elucidate these interactions.

## 4 DISCUSSION

Autophagy is a catabolic process of recycling specific cargoes, including misfolded proteins, damaged organelles, and metabolites in cells, by a double membrane vesicle designated AP followed by fusion with lysosomes for degradation. The energy and amino acids produced are then reused to maintain cellular homeostasis. In contrast to the conventional degradative autophagy, the secretory autophagy participating in cargo secretion via the type III UPS pathway is getting more attention.^2^, ^3^ Emerging evidence reveals that autophagy‐related exocytosis affects normal and pathologic physiology, including immune response, metabolism, senescence, cancer development, neurodegeneration, and pathogen infection.^19^, ^31^, ^32^, ^33^, ^34^, ^35^ Rab proteins function in vesicle transport, including endocytosis, vesicle transition, and exocytosis. Rab proteins regulate the initiation and termination of vesicle trafficking by cycling between GDP‐bound inactive Rab and GTP‐bound active Rab.^11^ The active GTP‐bound Rabs interact with specific effectors and recruit them to their locations for vesicle formation, movement, tethering, and fusion. Rab proteins regulate exocytosis by interacting with various effector proteins, including PI3Kγ, OCRL1, and TBK‐1.^21^ Many Rab proteins involved in regulating the autophagy process have been reported.^12^ We recently reported that secretory autophagy plays an enhancing role in the secretion of insulin and TIPM1 through a Rab37‐dependent process, indicating that it regulates secretory autophagy machinery. In addition, protein level and Rab37 activation (GTP form) determined the direction of vesicle trafficking.^22^ It implies that the activation of Rab protein is a determinant factor of Rab‐anchored vesicle trafficking. In addition, we reveal that the active form of Rab37 increases MAP1LC3/LC3 lipidation and is essential for promoting insulin secretion by autophagy.^6^ Altogether, Rab proteins play diverse roles in degradative and secretory autophagy. However, the underlying mechanisms of Rab proteins regulating secretory autophagy‐mediated cargo exocytosis remain poorly understood.

This study reveals that except Rab7a, five Rab proteins, Rab8a, Rab11b, Rab27a, Rab35, and Rab37, were highly expressed in the purified AP of lung cancer cells (CL1‐5‐Q89L) with a high secretory tendency (Q) (Figure 3, column 4 vs. column 3). Others have reported that Rab8a, Rab27a, and Rab37 are involved in secretory autophagy. Rab11 family proteins recycle proteins from endosomes to the plasma membrane, transporting molecules from the trans‐Golgi network to the plasma membrane and phagocytosis. Rab11b is primarily localized to the recycling compartment around the centriole and is an essential part of the vesicle machinery.^36^ In lung cancer cells, IFN‐γ triggered annexin A2 (ANXA2) exocytosis via a Rab11‐Rab8a and Rab27a mediated secretory autophagy pathway including the fusion of AP and MVB to form amphisome followed by migration to the plasmid membrane and exocytosis.^18^ Rab35 mediates the exosome release across the plasma membrane via the synaptosome‐associated protein mechanism.^37^ Inhibition of Rab35 function results in accumulating intracellular endosomal vesicles and impairs exosome secretion.^38^ In contrast, Rab7a was detected at a very low level in the AP fraction of CL1‐5‐Q89L cells with a high secretory tendency (Q) (Figure 3, row 6). Rab7a controls the AP maturation and autophagic flux, transfers cargos from MVBs to lysosomes, and participates in the regulation of degradative autophagy,^24^, ^39^ which is consistent with the low colocalization of Rab7 and LC3 puncta in Figure 2 and low content of Rab7a in the AP of the secretory tendency cells in Figure 3. Rab27a and Rab27b act in MVEs (multivesicular endosomes) docking at the plasma membrane. Silencing Rab27a increased the size of MVEs, whereas silencing Rab27b redistributed MVEs toward the perinuclear region. Rab27a is also responsible for transporting the amphisome to the plasma membrane. These two Rab27 isoforms function differently in the exosomal pathway.^19^, ^27^, ^39^ In summary, the above findings imply that Rab8a, Rab11b, Rab27a, Rab35, and Rab37 might participate in the secretory autophagy. Moreover, higher colocalization of Rab8a, Rab35, and Rab37 proteins with LC3 puncta in the cells with secretory tendency further sustains that these Rab proteins participate in the trafficking of secretory autophagic‐like vesicles in the cell (Figure 2A,C,E). Our findings and others' reports support that Rab8a, Rab11, Rab27a, and Rab37 are involved in autophagy‐related cargo secretion.^6^, ^18^, ^19^ Moreover, this is the first report that Rab35 may participate in the secretory autophagy (Figures 2E and 3). Our findings open a new avenue toward exploring these Rab proteins mediated cargo secretion through the secretory autophagy and warrant further identification of the effectors and specific cargoes regulated by these Rab proteins.

## CONFLICT OF INTEREST STATEMENT

The authors declare no conflicts of interest.
